# Supplementary material for: The Potential Role of the Methionine Aminopeptidase Gene PxMetAP1 in a Cosmopolitan Pest for Bacillus thuringiensis Toxin Tolerance
Source: Int J Mol Sci. 2022 Oct 27;23(21):13005. doi: 10.3390/ijms232113005 (PMC9656409; doi:10.3390/ijms232113005)
Supplement: Supplementary file 1 [file ijms-23-13005-s001.zip › ijms-1928965-supplementary.pdf]

International Journal of
Molecular Sciences

MDPI

Supplementary Material

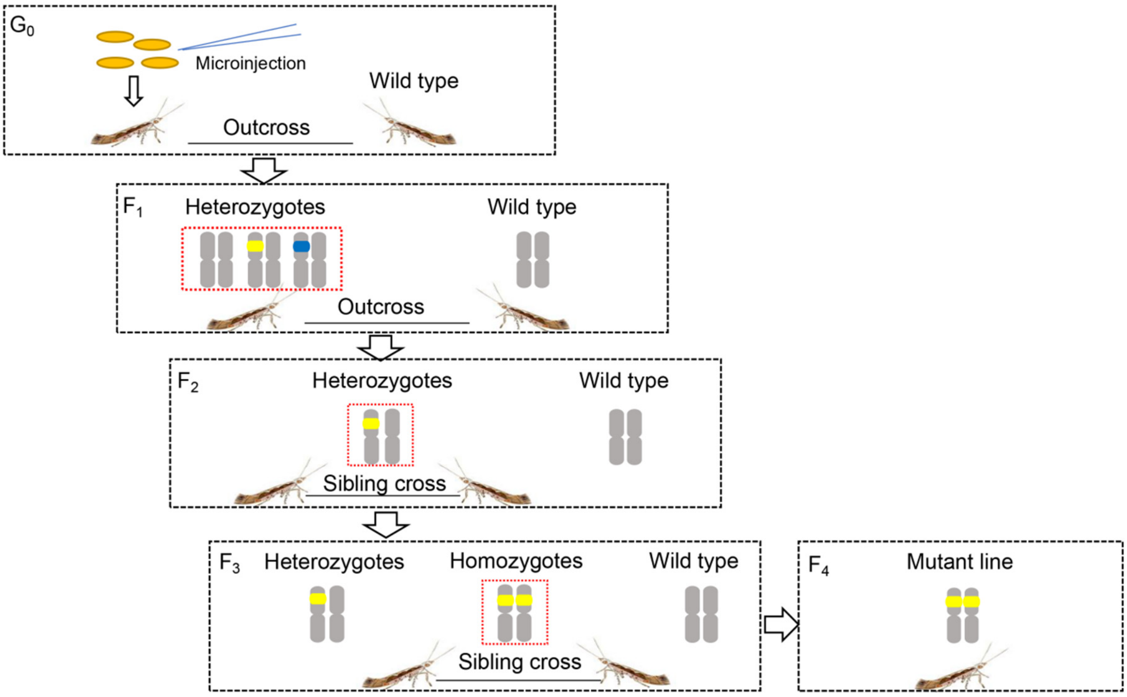

Figure S1. Flowchart of homozygous mutant strain screening. Each long box represents a single pair of male and female adults, the red box represents a single pair of parents whose mutations were identified by sequencing and retained for next-generation mating, and different colors represent different types of mutations.

Table S1. Inheritance of resistance between *PxMetAP1* gene and Cry1Ac in *P. xylostella*.

Backcross family [a]	Untreated control		Cry1Ac treated	
	N [b]	Larvae with $R_{M1}R_{M1}$ (%)	N [c]	Larvae with $R_{M1}R_{M1}$ (%)
BCa1	37	52.6	19	100
BCa2	43	45.9	20	100
BCa3	39	43.2	18	100
BCa4	45	47.7	22	100
BCa5	47	44.3	18	100
BCb1	44	54.1	23	100
BCb2	42	51.5	19	100
BCb3	36	48.4	24	100
BCb4	38	50.8	22	100
BCb5	46	46.7	21	100
Total	417	-	206	-

[a] Backcross family a (BCa1-5) was produced by an F_1 female with a Cry1S1000 male, and backcross family b (BCb1-5) was produced by an F_1 male with a Cry1S1000 female.
[b] Number of early 3rd instar larvae used for control (normal diet) and genotyped in each backcross family.
[c] Number of surviving early 3rd instar larvae in each backcross family that were genotyped for the treatment group (diet containing 0.5 μg/mL Cry1Ac toxin). 40 larvae were picked for each backcross family in the treatment group, for a total of 400 larvae.

Table S2. Susceptibility of different *P. xylostella* strains to Bt toxin.

Toxins	Strains	Slope (SE)	LC_{50} (μg/mL)	95% CI	RR[a]
Cry1Ac	G88	3.515 (0.382)	0.155	0.138-0.174	1
	G88-No-Indel	4.664 (0.569)	0.158	0.140-0.177	1.02
	PxMetAP1-KI-7	3.171 (0.269)	0.865	0.755-0.993	5.58
Cry1Ab	G88	2.292 (0.181)	1.966	1.618-2.395	1
	G88-No-Indel	1.932 (0.146)	2.040	1.653-2.516	1.04
	PxMetAP1-KI-7	2.401 (0.221)	6.781	5.593-8.121	3.45
Cry2Aa	G88	3.625 (0.375)	50.818	44.576-58.000	1
	G88-No-Indel	4.337 (0.453)	49.943	43.853-57.329	0.98
	PxMetAP1-KI-7	4.147 (0.419)	156.192	137.422-177.645	3.07
Cry2Ab	G88	3.578 (0.314)	12.563	8.267-17.958	1
	G88-No-Indel	3.310 (0.287)	13.102	9.111-17.985	1.04
	PxMetAP1-KI-7	2.885 (0.241)	28.448	20.599-37.992	2.26

[a] RR＝LC_{50} of each strain / LC_{50} of G88 strain
